# Do public healthcare programs make societies more equal? Cross-country evidence on subjective wellbeing

**DOI:** 10.1186/s13561-023-00467-2

**Published:** 2023-11-24

**Authors:** Ryan Joseph R. Dizon

**Affiliations:** 1 School of Business, Faculty of Business and Law, University of Wollongong, Northfields Ave Wollongong, Wollongong, NSW 2522 Australia; 2 Department of Economics, School of Social Sciences, Ateneo de Manila University, Quezon City, Philippines

**Keywords:** Public health, Subjective wellbeing inequality, Universal health coverage, I14, I31, I18

## Abstract

**Background:**

Universal health coverage (UHC) aims to provide quality healthcare services and safeguard the population from the financial burden of catastrophic health expenditure. Its primary objectives are to improve longevity and enhance overall quality of life. This study investigates the relationship between UHC and the inequality in subjective wellbeing (SWB) and explores whether public health programs can reduce social inequality. By employing SWB inequality as a measure, we go beyond the conventional income-centric approach to assess social inequality.

**Methods:**

The SWB data used in this study are derived from the repeated cross-sectional survey obtained from the European Values Study (EVS) and the World Values Survey (WVS). We adopt an observational study design and employ statistical techniques, such as ordinary least squares, Oaxaca–Blinder decomposition, and the recentered influence function (RIF). The RIF, in particular, allows us to characterise the entire distribution of SWB, rather than focusing on a single point.

**Results:**

UHC programs are negatively associated with SWB inequality (-0.070, significant at 5%). The negative association is observed at the 5^th^, 50^th^, and 75^th^ percentiles of the SWB distribution, whilst the association becomes positive at the 95^th^ percentile. UHC programs do not contribute to the SWB inequality gap.

**Conclusions:**

UHC programs exhibit an inequality-reduction property when the inequality is not severe or when countries are more equal. However, their effectiveness diminishes in the presence of extreme inequality. Health programs do not contribute to the existing SWB inequality gap between developed and developing countries. Strengthening the two dimensions of the UHC program (i.e., service coverage and financial protection) will ensure better health and wellbeing for all, and potentially foster a more equal and inclusive society.

## Introduction

Inequality refers to the unequal distribution of resources and opportunities amongst the different groups in the society. It can arise in income, gender, health, opportunities, and other life domains. The most common and universal measure of inequality is income [1] because data are readily available and often regarded as a reflection of success and economic growth. Wilkinson and Pickett [2] noted that income inequality is associated with several social issues like poor physical and mental health, low social trust, low social mobility, and crime.

Focusing on health inequality or the disparity in health amongst social groups, the World Health Organization (WHO) advocated the establishment of Universal Health Coverage (UHC) in response mainly to one of the 17 United Nations Sustainable Development Goals (Goal 3)—ensuring healthy lives and promoting the wellbeing of people. UHC programs have two main dimensions, namely service coverage and financial protection. These dimensions aim to enhance access to quality healthcare services and provide financial protection against catastrophic health expenditures. Without UHC programs, millions of people around the world would be at risk and become vulnerable to health shocks and poverty [3, 4]. The absence of public health programs not only burdens households with the cost of the illness but also exacerbates income inequality. This situation occurs due to potential income losses, reduced basic consumption, and disruption of standard of living [3].

The success of UHC programs is contingent upon two critical factors, namely the allocation of resources to the healthcare sector and the political environment within a country. Behera and Dash [5] conducted a study amongst South-East Asian countries to examine the relationship between health expenditure and healthcare goals, including health service coverage from UHC. Their findings revealed that increasing public health expenditure plays a crucial role in achieving faster progress towards UHC. Moreover, Behera and Dash [6] highlighted some recommendations for achieving UHC, including generating health-specific revenues, improving tax administration, and implementing effective usage of health budget. In the same vein, Pacek and Radcliff [7] concluded that the effectiveness of institutions significantly influences the health and wellbeing of individuals through public health programs. This impact is realised through the implementation of policies that address socioeconomic situations and align with the government’s priorities and effectiveness.

Many researchers have highlighted the limitations of using income alone as a measure of social inequality and have suggested for the inclusion of subjective wellbeing (SWB) distribution as a more comprehensive and relevant measure, particularly for policy making [8]. SWB inequality comprehensively captures relevant life domains that matter to individuals and society. Several economists have accepted the view that subjective wellbeing or happiness is a valid representation of an individual’s utility and a valuable tool for informing public policies [9].

The present study aims to unpack the relationship between universal health coverage (UHC) and subjective wellbeing (SWB) inequality. Healthier individuals tend to be more productive and have better opportunities for improving their lives. Therefore, it is consequently necessary to understand the nexus between UHC and SWB, as well as its distribution, to gain a comprehensive understanding of social wellbeing. We contribute to the body of knowledge by focusing on health—measured/represented by the UHC program—as a chief determinant of SWB inequality, which serves as a measure of social inequality.

## Background and literature review

### SWB inequality

Subjective wellbeing inequality is gaining attention in the literature as a better measure of social inequality due to its comprehensive nature and fewer theoretical issues compared to income inequality. SWB is a multi-faceted concept, and it captures the relevant domains of human life. Individuals tend to recognise and give weights to those domains in assessing their level of satisfaction in life as well as happiness. Moreover, happiness or individual utilities are comparable and may not be totally different when trying to compare an individual’s happiness with another [8]. Consequently, scale invariance and being universal are the key properties of SWB inequality.

The SWB concept comes with weaknesses as well. SWB data come from simple questions such as “all things considered, how satisfied are you with your life as a whole these days?” or “taking all things together, would you say you are: very happy, quite happy, not very happy, not at all happy?” (WVS and EVS). The possible responses to such life satisfaction survey question range from 1 (dissatisfied) to 10 (satisfied), whilst 1 (very happy) to 4 (not at all happy) for the happiness question. The data or information that can be gathered from such questions can suffer from possible measurement errors, inconsistency, and mechanical correlation bias between SWB levels and dispersion [8, 10].

The previously identified limitations concerning the use of SWB as a measure of inequality have been empirically addressed by some authors proving that it is a valid and a more robust measure of inequality. In the paper of Goff et al. [8], the authors proved that the correlations between SWB level and inequality are largely explained by true correlation and only a small portion is attributable to the mechanical relationship using three tests.^1^ In addition, Benjamin et al. [11] substantiated that the responses from the SWB questions reflect the individuals’ utility-maximising decision and reveal their level of utility—SWB is a valid representation of utility. Moreover, SWB responses of individuals encapsulate the information about their capabilities and opportunities [12] which are the essential elements in understanding and gauging the level of inequality in society. Lastly, regarding the issue of consistency, the same authors have proven that using the questions adapted by the WVS survey has produced the most consistent results in revealing people’s level of utility in comparison to other types of SWB questions found in different surveys.

### Subjective wellbeing: cardinal or ordinal

In using SWB data that represent the utility of individuals, Ferrer-i-Carbonell and Frijters [13] discussed the two main assumptions used in the literature, namely ordinality and cardinality. These assumptions have led to disagreements amongst researchers regarding the operationalisation of the data and the interpretation of results. To date, the literature on SWB has not reached a universal consensus on which assumption should strictly prevail in this line of research.

Ordinality and cardinality play an important role in determining the appropriate methods and tools for evaluating the SWB data. If ordinality is assumed, using average and standard deviation are not appropriate for measuring both the level and dispersion of SWB since they are designed for non-ordinal data [14]. The use of the median or other methods like the one proposed by Cowell and Flachaire [15] is recommended in dealing with ordinal data. However, the literature also accepts the assumption of cardinality in SWB research [16]. In fact, one of the pioneers of SWB research, Ruut Veenhoven, has recommended the use of the standard deviation of life satisfaction as a (better) measure of inequality [17]. Moreover, it is argued that the assumption of cardinality is essential when conducting cross-country analysis or comparison. It provides practical measurements that generate insights which are necessary for unpacking the complex concept of SWB [10]. Intuitively, if the aim is to understand the social context of inequality, attention should be given to the information about *how much* a society is better or worse off from one period to another or relative to another society/country [18].

Evidence from the literature has addressed the standing issues regarding the two assumptions. For instance, Goff et al. [8] examined the correlation between the level and dispersion of satisfaction in life using the mean and standard deviation respectively, and introduced a purely ordinal measure of dispersion in their paper. The authors used European Social Survey, World Values Survey, Gallup World Poll, and Gallup-Healthways Well-Being Index datasets. Their findings revealed a smaller correlation between satisfaction with life (SWL)/SWB level and dispersion when ordinal measure was used, but otherwise, the results were largely similar compared to when the standard deviation was employed. Furthermore, whether the variable was analysed as ordinal or cardinal data did not significantly impact the results when regressed at the individual or aggregate level [13, 19]; both approaches generated similar results [20].

Following the prevailing literature on SWB, there is merit in operationalising cardinality in this area of research because respondents of SWB surveys are typically aware of the boundedness of the scale (i.e., from 1 = worst to 10 = best). The respondents can position themselves on the scale, identifying their level of happiness or satisfaction in life. If respondents have a clear comprehension of the extreme sides of the bounded scale in SWB surveys, their responses can reflect the *extent or degree* of their SWB. In contrast, if the scale is unbounded, such as ranging from $- \infty$ to $+ \infty$, the responses may perhaps be strictly referring to the rank or order. Consequently, the assumption of cardinality is also a valid and necessary concept used in SWB research.

### Health and SWB inequality

UHC programs are the most powerful defence against health shocks, as they ensure access to quality healthcare services and offer financial protection [21]. Improvements in the two dimensions of the program—service coverage and financial protection dimensions—have produced positive effects on people’s SWB. This finding can be attributed to the enhanced protection by the program, which serves as a safety net against catastrophic health expenditures. It relieves people from the uncertainties of dealing with poor health and the associated financial burden. UHC programs increase life expectancy and improve people’s lives, whilst contributing to the reduction of poverty [22]. Furthermore, these health programs have also contributed to the improvement of individuals’ mental health and subjective wellbeing [23–28].

The absence of health insurance has been shown to cause lower levels of happiness or life satisfaction, as articulated in the literature [24]. Aside from the physical and mental agony caused by the health condition itself, the increasing healthcare costs are exacerbating the situation by draining the resources of patients and their family members. Such situations can lead to mental distress and make people less happy with life [17]. Furthermore, due to the skyrocketing healthcare costs, the probability of patients visiting healthcare providers is diminishing. The inaccessibility of healthcare services has been estimated in the literature to decrease the probability of being satisfied with life by more than 80 percent, and individuals are likely to report poor health status [24]. The incapacity of patients to consult health professionals due to cost issues has led to a 21 percent drop in a person’s happiness level, which is equivalent to being unemployed for a year [29]. Expanding on this perspective, if the patient’s or population’s health concerns—especially the mental aspect—are not properly addressed by health professionals, it can result in ill-social behaviours such as crime, excessive alcohol consumption, smoking, and non-adherence to treatments [30], which are detrimental to the society. These outcomes contribute to explaining the level of satisfaction individuals feel about their lives and highlighting the strong correlation between health and SWB. From a society’s standpoint, the different health statuses and other factors affecting people’s happiness or life satisfaction determine the distribution of SWB.

## Methods

### Data sources and variables

Following an observational study design, the data on subjective wellbeing (life satisfaction) are derived from the integrated EVS and WVS dataset (1990–2014) [31], which covers 77 countries (see Appendix 1). The repeated cross-sectional data set consists of approximately 506,000 individual respondents from different countries. The survey sample size ranges from 1200 to 1500. Data collection is conducted through face-to-face interviews at respondent’s residences. The two organisations have collaborated and worked closely to ensure uniformity in the variable structure across both surveys.

The complete list of variables as well as their definitions and sources are detailed in Table 1. All variables are expressed as country-level data (i.e., year averages). Panel A (Table 1) contains all the control variables in the model. In addition to the demographic variables, the study also includes ‘political position’ as one of the covariates in the model. Political position refers to whether countries are classified as liberal or conservative. It has been established in the literature that the political stance of individuals is related to their SWB. Previous studies have found that politically conservative countries or individuals tend to experience greater happiness and higher life satisfaction compared to liberals [32]. This outcome can be attributed to their tendency to practice positive adjustment, which results in fewer mental and emotional problems.

**Table 1:** Definition and Source

**Variable Name**	**Definition**	**Source/s**
Panel A: Control Variables		
Age	Weighted average age of the respondents	European Values Study and World Values Survey
Culture zone	The Culture zones are: 1. Reformed West, 2. New West, 3. Old West, 4. Returned West, 5. Orthodox East, 6. Indic East, 7. Islamic East, 8. Sinic East, 9. Latin America, and 10. Sub-Saharan Africa. Countries are classified based on their technological and democratic achievements	Rising Freedom by Christian Welzel
Economic status	Annual percentage growth rate of GDP at market prices based on constant local currency. Aggregates are based on constant 2010 U.S. dollars	World Development Indicators
Education	Education index is an average of mean years of schooling (of adults) and expected years of schooling (of children), both expressed as an index obtained by scaling with the corresponding maxima	United Nations (UNDP)
Effectiveness of the government	Government Effectiveness captures perceptions of the quality of public services, the quality of the civil service and the degree of its independence from political pressures, the quality of policy formulation and implementation, and the credibility of the government’s commitment to such policies. Estimate gives the country’s score in units of a standard normal distribution ranging from -2.5 to 2.5	World Development Indicators
GDP per capita	GDP per capita is gross domestic product divided by midyear population. Data are in constant 2010 U.S. dollars	World Development Indicators
Marital status	Ratio of respondents who are in a relationship (married and living together as married) over those who are not in a relationship (divorced, separated, widowed, single/never married, divorced/separated/widowed)	European Values Study and World Values Survey
Political position	Weighted average of respondents self-positioning in political scale. Possible responses ranging from 1 = left (liberal) to 10 = right (conservative)	European Values Study and World Values Survey
Sex ratio	Ratio of female respondents over male respondents	European Values Study and World Values Survey
Unemployment	Unemployment refers to the share of the labour force that is without work but available for and seeking employment	World Development Indicators
Panel B: Universal Health Coverage Indexes		
Out-of-pocket expenditure (Financial protection dimension)	Share of out-of-pocket payments of total current health expenditures. Out-of-pocket payments are spending on health directly out-of-pocket by households	World Development Indicators
Basic hospital access (Service coverage dimension)	Hospital beds (per 1,000 people)	World Health Organization
Full child immunization (Service coverage dimension)	Child immunization, Diphtheria-Pertussis-Tetanus (DPT), measures the percentage of children ages 12–23 months who received DPT vaccinations before 12 months or at any time before the survey. A child is considered adequately immunized against DPT after receiving three doses of vaccine	World Health Organization and United Nations International Children’s Emergency Fund
Prevention and treatment of raised blood glucose (Service coverage dimension)	Percent of defined population with fasting glucose ≥ 7.0 mmol/l or history of diagnosis with diabetes or use of insulin or oral hypoglycaemic drugs (crude estimate)	World Health Organization
Tuberculosis treatment (Service coverage dimension)	Treatment success rate	World Health Organization
Panel C: Outcome Variables		
Life satisfaction (standard deviation)	Life Satisfaction data are expressed as values ranging from 1 = dissatisfied to 10 = satisfied. Data is based on the country and wave/period	World Values Survey and European Values Study
Gini coefficient	Income inequality measure in the market (pre-tax, pre-transfer)	The Standardized World Income Inequality Database

*GDP* Gross domestic product

### UHC index

The WHO has come up with a standardised framework that is internationally recognised and used by countries/organisations in monitoring and developing the health programs. The framework has indicators that are used to gauge improvements in the healthcare provision and allow comparability across countries and over time. The 16 service coverage indicators are grouped into four categories: (1) reproductive, maternal, newborn and child health, (2) infectious diseases, (3) non-communicable diseases, and (4) service capacity and access (see Table 2). In addition to the service coverage dimension of UHC programs, another key aspect is the protection of people from catastrophic health expenses—financial protection dimension.

**Table 2:** Health Service Indicators

Reproductive, Maternal, Newborn, and Child Health	Infectious Diseases	Non-communicable Diseases	Service Capacity and Access
1. Family planning 2. Antenatal and delivery care 3. Full child immunization 4. Health-seeking behaviour for pneumonia	5. Tuberculosis treatment 6. Human Immunodeficiency Virus treatment 7. Use of insecticide-treated bed nets for malaria prevention 8. Adequate sanitation	9. Prevention and treatment of raised blood pressure 10. Prevention and treatment of raised blood glucose 11. Cervical cancer screening 12. Tobacco (non-) smoking	13. Basic hospital access 14. Health worker density 15. Access to essential medicines 16. Health security

Source: World Health Organization

To incorporate these two principal dimensions, we developed an index by selecting one indicator from each of the four categories outlined in the WHO framework. The selection of indicators was based on data availability from sources such as the WHO, WDI, and UN (refer to Panel B of Table1). The chosen indicators include full child immunisation, tuberculosis treatment, prevention and treatment of raised blood glucose, and basic hospital access to represent service coverage. Additionally, out-of-pocket expenditure was included to reflect the financial protection. To account for differences in units, the indices were converted into Z-scores and expressed in quintiles. The index (i.e., independent variable) was calculated using geometric mean and provides an assessment of the performance of UHC programs. Higher values in service coverage and financial protection indicate better-performing health programs. However, it is important to note that the UHC index does not capture the quality of services. For the main dependent variable (Panel C of Table 1), the study uses the distribution of SWB, measured by the standard deviation of life satisfaction, as the measure of social inequality.

### Statistical methods and empirical framework

This study uses the distribution of SWB and applies the unconditional quantile regression and decomposition technique known as the Oaxaca–Blinder (O–B) to empirically identify the dominant effect/s—endowment, coefficient, and interaction—in explaining the SWB inequality gap between developed and developing countries. In addition, the recentered influence function (RIF) method is employed to analyse unconditional partial effects on quantiles. The method is particularly suitable for studying inequality as it captures the entire distribution [33]. All estimations conducted in this paper were performed using Stata version 17.

OLS results are shown in Table 5. OLS, however, does not provide sufficient evidence for studying the relationship between UHC and SWB inequality because the effect of the covariates differs along the distribution of SWB [34–36]. We complement OLS with unconditional quantile regression (UQR) (see Table 6). The UQR, as established in applied economics literature, provides a better explanation as it determines the effect of the covariates across the entire distribution [37].

The literature typically classifies factors that influence as person’s SWB into two types: personal or individual characteristics and the characteristics of other individuals belonging to their reference group. Individuals often compare themselves to relevant others to assess their own wellbeing or to gain a sense of how they are doing compared to others. Building upon this rationality, Van Praag [18] concluded that a comprehensive understanding of SWB inequality requires considering the “referencing process.” Intuitively, without comparing oneself to a reference group, the concept of inequality would be incomprehensible both at the individual and societal levels.

The Oaxaca–Blinder (O–B) decomposition technique is employed in this study. This technique allows for the decomposition of the mean difference or gap between two groups of countries, taking into account the referencing process. The O–B decomposition approach helps in explaining the gap by attributing it to either the observed characteristics (explained component/endowment effect) or unequal opportunities (unexplained component/coefficient effect) as well as the possible interaction between the two components. Generally, the model is explained by the following equations:

1 $$\Delta \overline{Y }=\underbrace{\left({\beta }_{0}^{ {group}_{1}}-{\beta }_{0}^{ {group}_{2}}\right)}_{G_0}+\underbrace{\left({\beta }_{1}^{ {group}_{1} }{X}_{1}^{ {group}_{1} }- {\beta }_{1}^{ {group}_{2}}{X}_{1}^{{group}_{2}}\right)}_{G_1}+\underbrace{\left({\beta }_{2}^{{group}_{1} }{X}_{2}^{{group}_{1} }- {\beta }_{2}^{{group}_{2}}{X}_{2}^{{group}_{2}}\right)}_{G_2}$$

2 $$\Delta \overline{Y }= {G}_{0}+ {G}_{1 }+ {G}_{2},$$

where $\Delta \overline{Y }$ is the gap between the mean outcomes of the two groups, and ${X}^{ {group}_{1}}$ and ${X}^{ {group}_{2}}$ are vectors of explanatory variables expressed as averages. $\Delta \overline{Y }$ can be explained by three components namely, ${G}_{0}$,${G}_{1},$ and ${G}_{2}$. The first term refers to the differences in the intercept, followed by the difference in ${X}_{1}$ multiplied ${\beta }_{1}$, and the last term reflects the difference in ${X}_{2}$ multiplied by ${\beta }_{2}$. The decomposition of the total mean gap between the two groups can be expressed in two ways:

3 $$\Delta \overline{Y }= \Delta X {\beta }^{ {group}_{2}}+ \Delta \beta {X}^{{group}_{1}}$$

4 $$\Delta \overline{Y }= \Delta X {\beta }^{{group}_{1}}+ \Delta \beta {X}^{{group}_{2}}$$

where

$$\begin{array}{c}\Delta \overline{Y } = { Y}^{{group}_{1} }- {Y}^{{group}_{2}}\\ \Delta X = { X}^{{group}_{1} }- {X}^{{group}_{2}}\\ \Delta \beta = { \beta }^{{group}_{1}}- {\beta }^{{group}_{2}}\end{array}$$

In a more general case, the goal of identifying which effect/s (i.e., endowment, coefficient, or interaction) explains the gap is achieved by the decomposing and estimating its effects on the outcome. Consider the following equations:

5 $$\Delta \overline{Y } = \Delta X{\beta }^{{group}_{2}}+ \Delta \beta {X}^{{group}_{2}}+ \Delta X\Delta \beta$$

6 $$\Delta \overline{Y } = E+C+CE$$

The endowment, coefficient, and interactions effects are denoted by $E,C,$ and $CE$, respectively as shown in Eq. 6.

Unpacking the major drivers or contributors to happiness inequality, researchers have used the recentered influence function method (RIF) in their papers [38, 39]. This method is used to analyse the impact or to obtain partial effects of explanatory variables on unconditional quantile of the outcome variable. Similar to the O–B technique, the RIF decomposes the total change into two effects, namely the endowment and coefficient effects, providing a generalised version of O–B technique. Firpo et al. [40] established that the O–B technique can be used in combination with RIF to analyse the decomposition of any distribution.

We further extend the application of the O–B decomposition technique by combining it with the RIF to estimate the effect of each of the explanatory variable on the unconditional quantiles (percentile = 5, 25, 50, 75, 95). The two groups considered in this study are represented by countries classified as developing (Group 1) and developed (Group 2). The classification of countries is based on the framework of the United Nations Department of Economic and Social Affairs [41].

Lastly, to provide evidence regarding the relationship between a country’s level of technological and democratic developments and the distribution of SWB, an analysis is presented based on the 10 Culture Zone (CZ) framework developed by Christian Welzel [42]. The categorisation of countries into culture zones is determined by their technological and democratic achievements, which promote human empowerment. Based on the Human development index (HDI), the majority of the top-ranked countries are classified as developed and belong to CZs 1, 2, and 3. The HDI scopes three important domains of human development, namely health, knowledge, and standard of living. This finding supports the validity of study’s hypothesis that developed countries have a better healthcare system relative to developing ones—characterised by greater access to quality healthcare services and financial protection—in addition to other measures of human development. These factors significantly influence the SWB of the population and its distribution.

## Results

Combining the five country waves from the WVS and three from the EVS, the study uses 288 observations in the analysis spanning from 1990 to 2014 (see Appendix 1). The countries included in the study have participated in the survey for at least two waves.

As seen in Table 3, the global average for life satisfaction is 6.72 (out of 10), whilst the UHC index is 2.78 (out of 5), capturing both service coverage and financial protection. According to the SWB ranking of countries from the World Happiness Report [43], Nordic countries, namely Finland, Denmark, Sweden, Norway, and Iceland occupy the top positions, followed by other developed countries like New Zealand, Australia, Canada, United States, and United Kingdom. These developed countries have also been recognised to have the strongest public health systems in the world. Clustered by culture zone (CZ), countries belonging to CZ 1 have the highest life satisfaction (both mean and median), followed by CZ 2 (see Table 4). However, when it comes to the distribution of SWB, CZ 2 has the lowest standard deviation. These findings are consistent with the World Happiness Report, where most of the Nordic countries are clustered in CZ 1, and other developed countries such as Australia, Canada, New Zealand, and the United States belong to CZ 2. On the other hand, CZs 6 and 10 exhibit the highest levels of SWB inequality, with CZ 7 following closely.

**Table 3:** Descriptive statistics

**Variable**	**Mean**	**Median**	**Standard Deviation**	**Min**	**Max**
Life satisfaction (LS)	6.72	6.90	1.0	3.73	8.5
LS SD	2.14	2.14	-	1.21	3.35
UHC index	2.78	2.83	0.64	1.38	4.78
Age	42.79	43.86	4.40	30.68	53.34
Economic Status	3.49	3.65	4.945	-11.615	-1.719
Education	0.69	0.70	0.14	0.24	0.93
Effectiveness of the government	0.5600	0.5470	0.9387	-1.719	2.251
GDP per capita	18451.50	9607.83	19353.17	470.27	108577.40
Gini index	45.51	46.05	6.58	22.50	68.50
Marital status	1.84	1.68	0.77	0.60	6.54
Political position	5.61	5.57	0.59	3.80	9.09
Sex ratio	1.07	1.06	0.12	0.67	1.48
Unemployment	8.76	7.53	5.84	0.49	34.5

Refer to Table 1 for the variables’ definition and sources. The UHC index represents the level of service coverage and financial protection provided by the health program. Universal health coverage (UHC); Gross domestic product (GDP); Standard deviation (SD)

**Table 4:** Subjective wellbeing (life satisfaction) by culture zone

Culture Zone (CZ)	N	Mean	Median	Standard Deviation
1	35	7.75	7.76	0.22
2	14	7.61	7.66	0.11
3	48	7.25	7.24	0.19
4	37	6.40	6.39	0.19
5	55	5.56	5.56	0.19
6	19	6.57	6.70	0.34
7	22	6.01	5.93	0.33
8	11	6.63	6.53	0.20
9	34	7.50	7.46	0.26
10	13	6.25	6.25	0.34
Total	288			

Refer to Table 1 for the complete list/names of the culture zones. Countries are classified based on their technological and democratic achievements [42]. Refer to Appendix 2 for the list of countries and their respective culture zone

**Fig. 1:**
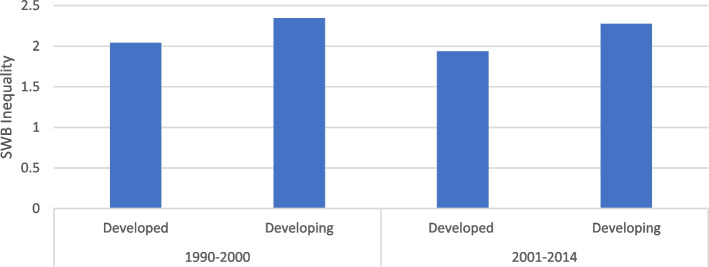
SWB inequality between developed and developing countries. Note: SWB (Subjective Wellbeing). SWB inequality is measured by standard deviation

Figure 1 shows that inequality is generally higher for developing countries (i.e., more unequal). This scenario is supported by the idea that typically developing countries have less economic opportunities, low social security, and less access to both quality education and healthcare services compared to developed ones [44].

**Fig. 2:**
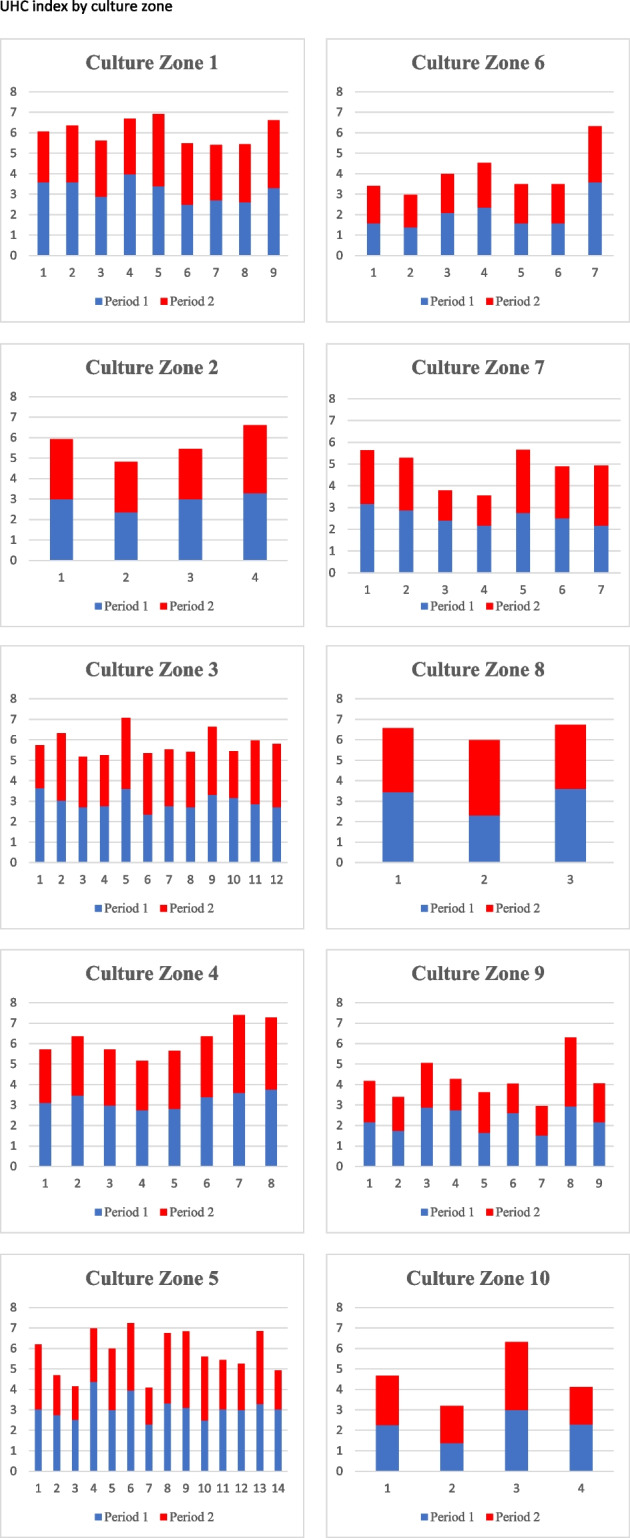
UHC index by culture zone. Note: The UHC index was calculated using data from the WHO, WDI, and UN databases (see Table 1). The vertical axis represents the UHC index, whilst the horizontal axis represents the number of countries belonging to a specific culture zone. Period 1 refers to the first wave/year in which a country was surveyed, and Period 2 refers to the latest wave/year. Refer to Appendix 2 for the list of countries and their respective culture zone

To provide more context, in addition to classifying countries as developed or developing, the study presents Fig. 2, which shows the variation in the UHC index from 77 countries categorised based on their respective culture zones. The figure also highlights the amount of variation observed in the data, suggesting that there is sufficient variation to establish the relationship between UHC and SWB inequality.

Table 5 presents evidence indicating a negative association between UHC programs and SWB inequality. The findings suggest that an increase in both service coverage and financial protection through UHC is associated with a reduction in SWB inequality by a standard deviation of 0.070. In addition, when examining income inequality—measured by Gini coefficient—the study finds a similar association as observed in SWB inequality; however, it is insignificant. Aside from UHC, the study also finds a negative correlation between SWB inequality and the countries’ economic status and GDP. Conversely, unemployment appears to have a positive correlation with the distribution of SWB.

**Table 5:** OLS results

Variables	Life Satisfaction Distribution (Standard Deviation)	Income Distribution (Gini)
UHC	-0.070** (0.027)	-0.650 (0.684)
Age	-0.001 (0.006)	0.171 (0.109)
Education	-0.131 (0.252)	3.702 (4.518)
Economic status	-0.006** (0.003)	-0.022 (0.068)
CZ2	0.111** (0.054)	0.155 (1.039)
CZ3	0.173*** (0.050)	1.667 (0.885)
CZ4	0.382*** (0.070)	0.317 (1.391)
CZ5	0.497*** (0.093)	0.307 (1.802)
CZ6	0.201* (0.118)	-1.513 (2.030)
CZ7	0.629*** (0.118)	-2.308 (1.783)
CZ8	0.202*** (0.077)	-3.623* (2.147)
CZ9	0.247*** (0.078)	5.677*** (1.529)
CZ10	0.521*** (0.133)	10.841*** (2.229)
GDP	-0.000* (0.000)	-0.000** (0.000)
Sex ratio	0.144 (0.127)	-9.375*** (3.516)
Marital status	0.023 (0.026)	-0.153 (0.480)
Unemployment	0.005* (0.003)	0.341*** (0.062)
Political position	0.010 (0.035)	-2.273*** (0.708)
Effectiveness of the government	0.111 (0.042)	4.319*** (0.926)
Prob > F	0.0000	0.0000
R-squared Observations	0.566 288	0.459 288

Refer to Table 1 for the definition of the variables. Ordinary least squares (OLS), universal health coverage (UHC), gross domestic product (GDP). ***p < 0.01, **p < 0.05, *p < 0.1 significance level at 1%, 5%, and 10%. Robust standard errors are in parentheses

In the analysis based on the 10 culture zones, the results indicate that all countries belonging to CZs 2 to 10 have a higher dispersion of SWB (i.e., a more unequal distribution) compared to countries belonging to CZ 1 (base category). All the results are statistically significant.

Using the unconditional quantile regression (UQR) approach, the results from Table 6 indicate significant variations and differential impacts of covariates that exist across different percentiles (i.e., distribution of SWB). UHC programs generally have a negative and significant association (except for the 25^th^ percentile) with SWB inequality from the 5^th^ to the 75^th^ percentile. Furthermore, the results indicate that unemployment and sex ratio have significant positive relationship with SWB inequality, whilst economic status, age, and effectiveness of the government show a significant negative correlation with the outcome variable. GDP also has negative correlation; however, the magnitude is almost negligible. Across all segments of the distribution, unemployment consistently demonstrates a positive relationship with SWB inequality. This finding suggests that higher rates of unemployment in a country are associated with increased inequality, which is particularly pronounced at the highest percentile.

**Table 6:** Unconditional quantile regression (SWB inequality)

**Percentile**	${{\varvec{R}}{\varvec{I}}{\varvec{F}}}_{5}$	${{\varvec{R}}{\varvec{I}}{\varvec{F}}}_{25}$	${{\varvec{R}}{\varvec{I}}{\varvec{F}}}_{50}$	${{\varvec{R}}{\varvec{I}}{\varvec{F}}}_{75}$	${{\varvec{R}}{\varvec{I}}{\varvec{F}}}_{95}$
Variables					
UHC	-0.148** (0.064)	-0.061 (0.044)	-0.136*** (0.039)	-0.093** (0.047)	0.137* (0.079)
Age	-0.003 (0.014)	0.009 (0.009)	-0.021** (0.008)	0.009 (0.010)	0.216 (0.017)
Education	0.524 (0.473)	-0.123 (0.321)	0.021 (0.285)	-0.440 (0.343)	-0.044 (0.581)
Economic status	-0.008 (0.007)	-0.001 (0.005)	-0.008* (0.004)	0.001 (0.005)	-0.030*** (0.008)
CZ2	0.90*** (0.174)	0.048 (0.118)	-0.051 (0.105)	-0.003 (0.126)	0.058 (0.214)
CZ3	0.923*** (0.132)	0.298*** (0.090)	0.026 (0.080)	-0.032 (0.096)	0.073 (0.162)
CZ4	0.983*** (0.182)	0.464*** (0.124)	0.396*** (0.110)	0.266** (0.132)	0.076 (0.224)
CZ5	1.00*** (0.235)	0.305* (0.159)	0.562*** (0.141)	0.600*** (0.170)	0.431 (0.288)
CZ6	0.678*** (0.254)	0.086 (0.172)	0.063 (0.153)	0.118 (0.184)	0.695** (0.311)
CZ7	1.00*** (0.240)	0.369** (0.162)	0.441*** (0.144)	0.841*** (0.173)	1.364*** (0.294)
CZ8	0.926*** (0.216)	0.092 (0.146)	0.030 (0.130)	0.029 (0.156)	0.237 (0.265)
CZ9	0.924*** (0.203)	0.280** (0.137)	0.174 (0.122)	0.121 (0.147)	0.478* (0.249)
CZ10	1.00*** (0.265)	0.370** (0.180)	0.282* (0.159)	0.588*** (0.192)	1.184*** (0.325)
GDP	-0.000 (0.000)	-0.000*** (0.000)	-0.000 (0.000)	0.000 (0.000)	0.000 (0.000)
Sex ratio	0.014 (0.359)	0.415* (0.243)	0.207 (0.216)	0.160 (0.260)	-0.394 (0.441)
Marital status	0.078 (0.057)	0.038 (0.038)	0.055 (0.034)	-0.034 (0.041)	-0.014 (0.070)
Unemployment	0.001 (0.006)	0.004 (0.004)	0.007* (0.004)	0.006 (0.005)	0.026*** (0.008)
Political position	0.015 (0.064)	-0.018 (0.044)	-0.023 (0.039)	0.045 (0.047)	0.109 (0.079)
Effectiveness of the government	0.018 (0.086)	-0.054 (0.058)	-0.013* (0.051)	0.057 (0.062)	0.117 (0.105)
Constant	0.757 (0.756)	1.033** (0.512)	3.030*** (0.455)	1.881*** (0.547)	0.697 (0.928)
R squared	0.24	0.41	0.55	0.33	0.25
Observations	288	288	288	288	288

Refer to Table 1 for the definition of the variables. Universal health coverage (UHC), gross domestic product (GDP). ***p < 0.01, **p < 0.05, *p < 0.1 significance level at 1%, 5%, and 10%. Robust standard errors are in parentheses. ${RIF}_{5}$ represents the lowest inequality whilst ${RIF}_{95}$ represents the highest inequality

In identifying the drivers of the inequality gap between developed and developing countries, we present the results from the O–B and O–B with RIF. Based on the O–B estimation results (Table 7), there is a significant overall difference between developed and developing countries. The significant difference is explained by the observed characteristics or the endowments (explained component). Factors like education, GDP, and effectiveness of the government appear to be significant drivers of the inequality gap in SWB.

**Table 7:** Oaxaca Blinder decomposition of SWB mean gap (SWB inequality gap**)**

Variables	Overall	Explained	Unexplained	Interaction
UHC		0.020 (0.022)	-0.016 (0.177)	0.003 (0.031)
Age		-0.039 (0.038)	-1.210** (0.511)	0.149** (0.064)
Education		0.075* (0.039)	0.484 (0.324)	-0.106 (0.072)
Economic status		-0.012 (0.012)	0.005 (0.012)	0.006 (0.015)
GDP		0.087* (0.046)	-0.133 (0.267)	0.010 (0.221)
Sex ratio		-0.002 (0.008)	0.415 (0.321)	-0.019 (0.016)
Marital status		-0.002 (0.006)	0.090 (0.079)	0.011 (0.011)
Unemployment		0.001 (0.009)	0.122** (0.049)	0.027* (0.016)
Political position		0.011 (0.019)	-0.349 (0.334)	-0.023 (0.023)
Effectiveness of the government		0.303*** (0.055)	0.206*** (0.075)	-0.234* (0.086)
**Overall**				
Group 1 (developing)	2.296*** (0.027)			
Group 2 (developed)	1.992*** (0.024)			
Difference	0.304*** (0.036)			
Explained	0.442*** (0.068)			
Unexplained	-0.061 (0.160)			
Interaction	-0.077 (0.171)			
Observation	288			

Refer to Table 1 for the definition of the variables. Universal health coverage (UHC), gross domestic product (GDP). ***p < 0.01, **p < 0.05, *p < 0.1 significance level at 1%, 5%, and 10%. Robust standard errors are in parentheses

Focusing on the UHC program, the O–B decomposition technique indicates that the public health program is not a significant driver of the existing SWB inequality gap between the two classifications of countries. UHC programs are not contributing to the SWB inequality gap between the developed and developing countries. Moreover, the negative correlation observed in the OLS and UQR estimations (Tables 5 and 6) provide further evidence supporting the idea that UHC programs are a significant tool in improving people’s SWB and its distribution.

The results from the O–B with RIF estimation (Table 8) provide a more comprehensive analysis of SWB inequality across the entire distribution. Unlike the O–B estimation, which focuses on the mean-based point in the distribution, the O–B with RIF estimation examines the unconditional partial effects on quantiles, allowing for a more nuanced understanding of the relationship. This approach adds valuable context and information to the discussion, allowing for a more robust interpretation of the results that is critical for policy making.

**Table 8:** Oaxaca–Blinder decomposition of SWB inequality gap with RIF

**Percentile Variables**	${{\varvec{R}}{\varvec{I}}{\varvec{F}}}_{5}$		${{\varvec{R}}{\varvec{I}}{\varvec{F}}}_{25}$		${{\varvec{R}}{\varvec{I}}{\varvec{F}}}_{50}$		${{\varvec{R}}{\varvec{I}}{\varvec{F}}}_{75}$		${{\varvec{R}}{\varvec{I}}{\varvec{F}}}_{95}$	
	**EX**	**UN**	**EX**	**UN**	**EX**	**UN**	**EX**	**UN**	**EX**	**UN**
UHC	0.066 (0.048)	0.447 (0.291)	0.009 (0.033)	-0.339* (0.203)	0.009 (0.027)	-0.097 (0.177)	-0.010 (0.031)	-0.083 (0.195)	-0.030 (0.057)	0.010 (0.391)
Age	0.071 (0.100)	-0.143 (0.993)	-0.055 (0.070)	-1.831*** (0.695)	-0.009 (0.057)	-0.974 (0.612)	0.006 (0.065)	0.061 (0.672)	-0.116 (0.121)	-1.905 (1.363)
Education	0.069 (0.104)	0.802 (0.502)	0.064 (0.072)	0.691** (0.351)	0.103* (0.060)	0.567* (0.309)	0.071 (0.067)	0.090 (0.339)	0.014 (0.125)	0.070 (0.687)
Economic status	-0.018 (0.030)	0.067 (0.067)	0.004 (0.021)	-0.015 (0.047)	-0.025 (0.018)	0.058 (0.041)	-0.024 (0.020)	0.022 (0.045)	0.021 (0.036)	-0.083 (0.089)
GDP	0.120 (0.085)	0.043 (0.079)	0.147** (0.060)	-0.064 (0.056)	0.069 (0.049)	-0.064 (0.052)	0.062 (0.055)	-0.065 (0.056)	0.039 (0.102)	0.050 (0.119)
Sex ratio	-0.006 (0.024)	0.061 (0.747)	-0.019 (0.017)	0.332 (0.507)	-0.013 (0.014)	0.236 (0.451)	-0.005 (0.015)	0.069 (0.493)	0.070** (0.034)	1.343 (1.011)
Marital status	-0.003 (0.016)	0.199 (0.191)	-0.002 (0.011)	0.183 (0.134)	-0.013 (0.011)	0.116 (0.117)	0.006 (0.011)	-0.103 (0.129)	-0.001 (0.019)	0.073 (0.258)
Unemployment	0.024 (0.024)	-0.053 (0.132)	-0.003 (0.016)	0.189** (0.093)	-0.005 (0.013)	0.147* (0.079)	0.006 (0.015)	0.089 (0.088)	0.009 (0.027)	0.110 (0.171)
Political position	0.058 (0.045)	-1.057 (0.793)	-0.017 (0.030)	-0.381 (0.552)	-0.003 (0.025)	-0.367 (0.469)	0.021 (0.029)	-0.266 (0.524)	0.072 (0.054)	-0.675 (1.018)
Effectiveness of the gov’t	-0.064 (0.126)	0.005 (0.021)	0.070 (0.087)	0.008 (0.015)	0.334*** (0.074)	-0.032* (0.795)	0.478*** (0.085)	-0.059** (0.023)	0.525*** (0.154)	-0.055 (0.034)
Constant		0.511 (1.332)		1.340 (0.928)		0.307 (0.016)		-0.060 (0.884)		-0.819 (1.733)
**Overall**										
Group1 (developing)	1.805*** (0.045)		2.127*** (0.036)		2.310*** (0.031)		2.466*** (0.032)		2.844*** (0068)	
Group2 (developed)	1.570*** (0.040)		1.814*** (0.030)		1.967*** (0.029)		2.160*** (0.035)		2.484*** (0.052)	
Difference	0.235*** (0.061)		0.312*** (0.047)		0.343*** (0.043)		0.306*** (0.048)		0.360*** (0.085)	
Explained	0.322** (0.148)		0.198* (0.195)		0.446*** (0.087)		0.610*** (0.100)		0.603*** (0.180)	
Unexplained	-0.087 (0.159)		0.114 (0.111)		-0.103 (0.092)		-0.305*** (0.104)		-0.243 (0.196)	

Refer to Table 1 for the definition of the variables. Universal health coverage (UHC), gross domestic product (GDP), EX means explained, and UN means unexplained. ***p < 0.01, **p < 0.05, *p < 0.1 significance level at 1%, 5%, and 10%. Robust standard errors are in parentheses. ${RIF}_{5}$ represents the lowest inequality whilst ${RIF}_{95}$ represents the highest inequality

The results suggest that there is a significant difference or gap between developed and developing countries when it comes to SWB inequality. More precisely, the positive coefficients indicate that developed countries are in a more progressive/advanced position compared to developing ones. Consistent with the previous O–B estimation, O–B with RIF results also show that UHC is not a significant driver. However, at the 25^th^ percentile, there is a significant negative association between UHC and SWB inequality in the unexplained part. Although significant, this effect can be ignored as the overall model indicates a statistically insignificant outcome for the unexplained component. At the midpoint of the distribution, the effectiveness of the government is identified as a significant driver up to the highest percentile (most unequal), but not in the lowest percentiles. This finding validates that more equal countries (developed) have better-performing governments (i.e., non-contributory to the gap), but the more unequal countries have otherwise. Lastly, the analysis reveals that a higher females-to-males sex ratio is a significant driver at the highest percentile, indicating the presence of gender inequality amongst the most unequal countries.

## Discussion

Understanding and addressing the negative impacts of social inequality is a complex and ongoing challenge, especially in developing countries and to some extent in certain developed societies. Despite the ongoing efforts, the problems arising from inequality continue to affect our society. One possible reason or factor contributing to this enduring challenge is the limited availability of comprehensive tools for assessing and understanding the true context and extent of the issue. The previous literature on inequality predominantly relied on income as a measure of social inequality, and this practice has limited our understanding of the problem. Certain types of inequality merit more attention, whilst other types may not warrant attention because they are not perceived as social problems since they come from personal choices. These nuances cannot be captured solely by income, highlighting the need for better tools such as SWB to provide a more comprehensive assessment.

The present study uses SWB inequality as a measure of social inequality and investigates its relationship with UHC. Our estimation results indicate that UHC programs serve as effective public policies in addressing not only people’s health-related concerns but also broader social issues, including social inequality as seen through the lens of SWB.

UHC programs demonstrate an inequality-reduction property. This finding is supported by the evidence from the OLS and UQR estimation results, showing a negative relationship between UHC and SWB inequality. However, this relationship does not hold true when there is extreme inequality in the society, as observed in the UQR results presented in Table 6. Specifically, at the 95^th^ percentile, the relationship becomes positive. This outcome suggests that the health program’s inequality-reduction property can lead to significant improvements at the lower end of the distribution—where countries are more equal. Conversely, at the 95^th^ percentile, representing the most unequal countries in the sample, UHC is positively correlated with SWB inequality.

The potential inequality-reduction effect of UHC programs in the lower part of the distribution can be explained by the tendency of more equal countries to prioritise minimising inequalities in the society. These (developed) countries are typically characterised by higher incomes, better life expectancy, and improved access to healthcare and opportunities. They are more inclined to implement policies, such as UHC, that aim to reduce disparities and improve the wellbeing of their population [45]. On the other side of the distribution (most unequal), UHC loses its inequality-reduction property because individuals at the top of the social class tend to benefit from the existing inequalities. This situation makes it more challenging and requires substantial effort to bring about significant changes that will benefit the entire society. In addition, in highly divided societies, there is typically less social trust, higher levels of corruption, and people tend to be less supportive of each other [46]. These scenarios are often observed in unequal society or developing countries where resources for providing quality public healthcare services are limited. As a result, people who have access to quality healthcare services can improve their health, whilst those with less or no access to healthcare services are left untreated, marginalised, and less satisfied with their lives. The unequal opportunities further contribute to societal inequality, leading to a significant positive correlation at the 95^th^ percentile.

Moreover, it is important to discuss the consistent relationship between unemployment and SWB inequality, particularly the more pronounced relationship observed at the 95^th^ percentile (significant at 1%), as evident from the UQR results. Intuitively, this outcome implies that in the most unequal societies, the impact of unemployment on SWB inequality is more severe, resulting in a significant widening of the gap within the society or country.

From the significant SWB gap identified between developed and developing countries, the O–B decomposition technique (Table 7) indicates that education, GDP, and the effective of the government make significant contributions to the existing gap between these two country classifications, whereas UHC does not. The empirical evidence aligns with the notion that developed/industrialised countries are typically more technologically advanced (e.g., education), more progressive based on productivity (e.g., income), and have efficient and people-serving governments—welfare states [7]. In contrast, developing countries face inherent disparities in these areas, leading to lower levels of SWB and higher SWB inequality. Consequently, it is expected that developing countries will lag behind developed ones.

Undoubtedly, the conceptualisation and implementation of UHC programs within countries represent one of the most significant public policies. These public health programs play a crucial role in serving the population's health and wellbeing, as well as fostering a more equal society. By providing access to essential healthcare services for all, these programs aim to reduce health disparities and promote equality. Notwithstanding the benefits realised from the health programs, further efforts are needed to fully maximise their potential and enhance the *coverage and quality* of service provided to people.

The need to further develop the health programs is suggested by the OLS results, which indicate a negative but insignificant relationship between UHC and income distribution as measured by the Gini coefficient. The lack of significance may suggest that the two dimensions of the public health programs are not sufficiently effective in addressing health issues and associated costs to improve income distribution within a country. The literature establishes a strong inverse relationship between income inequality and population health [47]. Many people are pushed into poverty, especially when they experience deteriorating health conditions, compounded by the global increase in healthcare costs. According to the WHO and the World Bank [48], approximately half of the world’s population does not have healthcare coverage, and 800 million people continue to spend more than 10 percent of their annual budget on healthcare. These individuals, who are both financially disadvantaged and in poor health, are unable to improve their health status and standard of living, which consequently impacts their productivity and earning capacity leading to lower incomes.

Putting together the pieces of evidence from the present study, we can understand that effective governance plays a crucial role in reducing SWB inequality in the society. When people have trust and confidence in the government’s ability to fulfill its responsibilities and serve its people, it can have a positive impact on reducing inequality. Individuals who benefit from good governance and experience the advantages of effective public policies (e.g., UHC) feel more secure and confident in making life decisions, which in turn contributes to improving people’s health and life satisfaction. To support this idea, a study in Japan found that in order to achieve parallel improvement with economic growth and people’s wellbeing, the establishment of a system of social safety nets is necessary to improve people’s life satisfaction [49].

The quality of institutions is one key element in ensuring social equality from the SWB perspective. The provision of healthcare, education, employment, and a safe environment are some of the commonly cited supports that well-performing governments provide their people. The realisation of government support is the main reason why Nordic countries have consistently been ranked as the happiest countries in the World Happiness Report. These factors are also present in developed countries such as Australia, New Zealand, Netherlands, and Switzerland.

## Limitations of the study

Although this study provides valuable insights into the (SWB) inequality space, it is important to acknowledge certain limitations that may affect the generalisability of the findings. First, the service indicators considered in the UHC index were limited by data availability and did not account for the quality dimension associated with the services provided in the health program. Second, the decomposition technique did not include culture zone as a control variable due to a lack of variation. The lack of variation may be attributed to the categorisation of countries into developed and developing groups, which is necessary for the decomposition process, along with the inclusion of 10 culture zone categorical variables—resulting in an unbalanced distribution or categorisation in the estimation. This limitation can be conceptually minimised by recognising the potential overlap in classifying countries as developed or developing and by categorising countries into their respective culture zones. Both processes use indicators related to economic growth and human development. Therefore, the exclusion of culture zone as a control variable may not introduce significant bias in the estimation. Lastly, the interpretations of the results, based on a repeated cross-sectional dataset, focus on within and between country variations and do not imply causality.

## Conclusions

The literature on inequality has evolved from using income as a measure of social inequality to focusing on SWB inequality as a comprehensive measure. The goal in this line of research is to attain social cohesion, alleviate the negative impacts of having unequal societies, and most importantly preserve human dignity, especially amongst the marginalised people in society.

The results of the study are pragmatic. UHC programs can potentially solve some social issues and make society more equal by promoting healthy lives and wellbeing for all—increased access to quality healthcare services and protection from catastrophic health expenditure. Since UHC programs can affect both health and non-health outcomes (e.g., self-confidence, social participation, knowledge, and labour market participation) [50, 51], it is intuitive to think that public health programs positively influence the important life domains of people and thus improve their life satisfaction.

This discussion is important as it recognises a better measure of inequality and unpacks the relationship between UHC and SWB inequality, which is scarcely discussed in the literature. Acknowledging the global trend, the next phase in this line of research is to find a clearer path or mechanism on how policies will help achieve the full potential of public health programs in improving people’s health and wellbeing as well as making societies more equal.

## Data Availability

The data sets used and analysed during the current study are available from the corresponding author upon reasonable request. The data are publicly available from the integrated European Values Study and World Values Survey, World Bank, World Development Indicators, World Health Organization, United Nations Development Programme, The Standardized World Inequality Database, and Rising Freedom (by Christian Welzel).

## Appendix 1

**Table 9:** List of countries and country waves

Albania (1998)	Estonia (2011)	Macedonia (1998)	Slovakia (1990)
Albania (2002)	Finland (1990)	Macedonia (2001)	Slovakia (1991)
Albania (2008)	Finland (1996)	Macedonia (2008)	Slovakia (1998)
Algeria (2002)	Finland (2000)	Malaysia (2006)	Slovakia (1999)
Algeria (2013)	Finland (2005)	Malaysia (2012)	Slovakia (2008)
Armenia (1997)	Finland (2009)	Malta (1991)	Slovenia (1992)
Armenia (2008)	France (1990)	Malta (1999)	Slovenia (1995)
Armenia (2011)	France (1999)	Malta (2008)	Slovenia (1999)
Argentina (1991)	France (2006)	Mexico (1990)	Slovenia (2005)
Argentina (1995)	France (2008)	Mexico (1995)	Slovenia (2008)
Argentina (1999)	Georgia (1996)	Mexico (1996)	Slovenia (2011)
Argentina (2006)	Georgia (2008)	Mexico (2000)	South Africa (1990)
Argentina (2013)	Georgia (2009)	Mexico (2005)	South Africa (1996)
Australia (1995)	Germany (1990)	Mexico (2012)	South Africa (2001)
Australia (2005)	Germany (1997)	Moldova (1996)	South Africa (2006)
Australia (2012)	Germany (1999)	Moldova (2002)	South Africa (2013)
Austria (1990)	Germany (2006)	Moldova (2006)	Spain (1990)
Austria (1999)	Germany (2008)	Moldova (2008)	Spain (1995)
Austria (2008)	Germany (2013)	Morocco (2001)	Spain (1999)
Azerbaijan (1997)	Ghana (2007)	Morocco (2007)	Spain (2000)
Azerbaijan (2011)	Ghana (2012)	Morocco (2011)	Spain (2007)
Bangladesh (1996)	Greece (1999)	Netherlands (1990)	Spain (2008)
Bangladesh (2002)	Greece (2008)	Netherlands (1999)	Spain (2011)
Belarus (1990)	Hungary (1991)	Netherlands (2006)	Sweden (1990)
Belarus (1996)	Hungary (1998)	Netherlands (2008)	Sweden (1996)
Belarus (2000)	Hungary (1999)	Netherlands (2012)	Sweden (1999)
Belarus (2008)	Hungary (2008)	New Zealand (1998)	Sweden (2006)
Belarus (2011)	Hungary (2009)	New Zealand (2004)	Sweden (2009)
Belgium (1990)	Iceland (1990)	New Zealand (2011)	Sweden (2011)
Belgium (1999)	Iceland (1999)	Nigeria (1990)	Switzerland (1996)
Belgium (2009)	Iceland (2009)	Nigeria (1995)	Switzerland (2007)
Bosnia and Herzegovina (1998)	India (1990)	Nigeria (2000)	Switzerland (2008)
Bosnia and Herzegovina (2001)	India (1995)	Nigeria (2011)	Thailand (2007)
Bosnia and Herzegovina (2008)	India (2001)	Norway (1990)	Thailand (2013)
Brazil (1991)	India (2006)	Norway (1996)	Trinidad and Tobago (2006)
Brazil (2006)	India (2014)	Norway (2007)	Trinidad and Tobago (2011)
Brazil (2014)	Indonesia (2001)	Norway (2008)	Turkey (1990)
Bulgaria (1991)	Indonesia (2006)	Pakistan (1997)	Turkey (1996)
Bulgaria (1997)	Iran (2000)	Pakistan (2001)	Turkey (2001)
Bulgaria (1999)	Iran (2007)	Pakistan (2012)	Turkey (2007)
Bulgaria (2005)	Iraq (2004)	Peru (1996)	Turkey (2009)
Bulgaria (2008)	Iraq (2006)	Peru (2001)	Turkey (2011)
Canada (1990)	Iraq (2012)	Peru (2006)	Ukraine (1996)
Canada (2000)	Ireland (1990)	Peru (2012)	Ukraine (1999)
Canada (2006)	Ireland (1999)	Philippines (1996)	Ukraine (2006)
Chile (1990)	Ireland (2008)	Philippines (2001)	Ukraine (2008)
Chile (1996)	Italy (1990)	Philippines (2012)	Ukraine (2011)
Chile (2000)	Italy (1999)	Poland (1990)	United Kingdom (1990)
Chile (2006)	Italy (2005)	Poland (1997)	United Kingdom (1998)
Chile (2011)	Italy (2009)	Poland (1999)	United Kingdom (1999)
Colombia (1997)	Japan (1990)	Poland (2005)	United Kingdom (2005)
Colombia (1998)	Japan (1995)	Poland (2008)	United Kingdom (2008)
Colombia (2005)	Japan (2000)	Poland (2012)	United Kingdom (2009)
Colombia (2012)	Japan (2005)	Portugal (1990)	Unites Stated (1990)
Croatia (1996)	Japan (2010)	Portugal (1999)	Unites Stated (1995)
Croatia (1999)	Jordan (2001)	Portugal (2008)	Unites Stated (1999)
Croatia (2008)	Jordan (2007)	Romania (1993)	Unites Stated (2006)
Cyprus (2006)	Jordan (2014)	Romania (1998)	Unites Stated (2011)
Cyprus (2008)	Korea, Republic (1990)	Romania (1999)	Uruguay (1996)
Cyprus (2011)	Korea, Republic (2001)	Romania (2005)	Uruguay (2006)
Czech Republic (1991)	Korea, Republic (2005)	Romania (2008)	Uruguay (2011)
Czech Republic (1998)	Korea, Republic (2010)	Romania (2012)	Venezuela (1996)
Czech Republic (1999)	Kyrgyzstan (2003)	Russia (1990)	Venezuela (2000)
Czech Republic (2008)	Kyrgyzstan (2011)	Russia (1995)	Vietnam (2001)
Denmark (1990)	Latvia (1990)	Russia (1999)	Vietnam (2006)
Denmark (1999)	Latvia (1996)	Russia (2006)	
Denmark (2008)	Latvia (1999)	Russia (2008)	
Egypt (2001)	Latvia (2008)	Russia (2011)	
Egypt (2008)	Lithuania (1990)	Rwanda (2007)	
Egypt (2013)	Lithuania (1997)	Rwanda (2012)	
Estonia (1990)	Lithuania (1999)	Serbia (1996)	
Estonia (1996)	Lithuania (2008)	Serbia (2001)	
Estonia (1999)	Luxembourg (1999)	Serbia (2005)	
Estonia (2008)	Luxembourg (2008)	Serbia (2008)	

## Appendix 2

**Table 10:** List of countries and their culture zone

**Culture Zone 1** **Reformed West**	**Culture Zone 2** **New West**	**Culture Zone 3** **Old West**	**Culture Zone 4** **Returned West**	**Culture Zone 5** **Orthodox East**
Denmark Finland Germany Iceland Netherlands Norway Sweden Switzerland United Kingdom	Australia Canada New Zealand United States	Andorra Austria Belgium Cyprus Estonia France Greece Ireland Israel Italy Luxemburg Malta Portugal Spain	Czech Republic Croatia Estonia Hungary Latvia Lithuania Poland Slovakia Slovenia	Azerbaijan Albania Armenia Belarus Bosnia Bulgaria Georgia Kyrgyzstan Macedonia Moldova Romania Russia Serbia Ukraine
**Culture Zone 6** **Indic East**	**Culture Zone 7** **Islamic East**	**Culture Zone 8** **Sinic East**	**Culture Zone 9** **Latin America**	**Culture Zone 10** **Sub-Saharan Africa**
Bangladesh India Indonesia Malaysia Pakistan Philippines Singapore Thailand	Algeria Egypt Iran Iraq Jordan Morocco Saudi Arabia Turkey	China Hong Kong Japan South Korea Taiwan Vietnam	Argentina Brazil Chile Colombia Dominican Rep Guatemala El Salvador Mexico Peru Trinidad and Tobago Uruguay Venezuela	Burkina Faso Ghana Mali Nigeria Rwanda Tanzania South Africa Uganda Zambia Zimbabwe

## Abbreviations

CZ: Culture Zone
EVS: European Values Study
GDP: Gross Domestic Product
HDI: Human Development Index
IO: Inequality of Opportunity
O–B: Oaxaca–Blinder
OLS: Ordinary Least Squares
RIF: Recentered Influence Function
SD: Standard Deviation
SWB: Subjective Wellbeing
SWL: Satisfaction with Life
UHC: Universal Health Coverage
UN: United Nations
UQR: Unconditional Quantile Regression
WDI: World Development Indicators
WHO: World Health Organization
WVS: World Values Survey

## Footnotes

1 The tests include a measure of aversion to inequality comparing between inequality-averse subjects and other subjects. Second, directly controlling for the mechanical correlation using social trust variable. Lastly, measuring a bound on the size of the mechanical correlation. For full discussion, see Goff et al., 2018.

## References

[1] Atkinson A, Bourguignon F. Handbook of income distribution. Elsevier. Amsterdam; 2000. https://www.inet.ox.ac.uk/publications/handbook-of-income-distribution-vol-1/.

[2] Wilkinson RG, Pickett KE (2009). Income inequality and social dysfunction. Annu Rev Sociol.

[3] Rashad AS. The catastrophic economic consequences of illness and their effect on poverty estimates in Egypt, Jordan, and Palestine. Working Paper Series No.842, The Economic Research Forum; 2014. https://ideas.repec.org/p/erg/wpaper/842.html.

[4] Ekman B (2007). Catastrophic health payments and health insurance: some counterintuitive evidence from one low-income country. Health Policy.

[5] Behera DK, Dash U (2020). Is health expenditure effective for achieving healthcare goals? Empirical evidence from South-East Asia Region. Asia Pac J Reg Sci.

[6] Behera DK, Dash U (2019). Impact of macro-fiscal determinants on health financing: empirical evidence from low-and middle-income countries. Glob Health Res Policy.

[7] Pacek A, Radcliff B (2008). Welfare policy and subjective well-being across nations: an individual-level analysis assessment. Soc Indic Res.

[8] Goff L, Helliwell J, Mayraz G (2018). Inequality of subjective well-being as a comprehensive measure of inequality. Econ Inq.

[9] Graham C (2008). Happiness and health: lessons and questions for public policy. Health Aff.

[10] Gluzmann P, Gasparini L. International inequality in subjective well-being: an exploration with the gallup world poll. Rev Dev Econ. 2017. 10.1111/rode.12356.

[11] Benjamin D, Heffetz O, Kimball MS, Rees-Jones A. Do people seek to maximize happiness? Evidence from new surveys. NBER working paper no. 16489, National Bureau of Economic Research; 2010. https://www.nber.org/system/files/working_papers/w16489/w16489.pdf.

[12] Graham C, Nikolova M (2015). Bentham or Aristotle in the development process? An empirical investigation of capabilities and subjective well-being. World Dev.

[13] Ferrer-i-Carbonell A, Frjiters P (2004). How important is methodology for the estimates of the determinants of happiness?. Econ J.

[14] Grimes A, Jenkins S, Tranquilli F. The relationship between subjective wellbeing and subjective wellbeing inequality: taking ordinality and skewness seriously. Motu Working Paper No. 20 – 09, Economic & Public Policy Research; 2020. https://motu-www.motu.org.nz/wpapers/20_09.pdf.

[15] Cowell F, Flachaire E (2017). Inequality with ordinal data. Economica.

[16] Easterlin RA (2006). Life cycle happiness and its sources: intersections of psychology, economics, and demography. J Econ Psychol.

[17] Okulicz-Kozaryn A, Mazelis JM (2017). More unequal in income, more unequal in wellbeing. Soc Indic Res.

[18] Van Praag B. Well-being inequality and reference groups: an agenda for new research. J Econ Inequal. 1995. 10.1007/s10888-010-9127-2.

[19] Diener E, Diener M, Diener C (1995). Factors predicting the subjective well-being of nations. J Pers Soc Psychol.

[20] Frey BS, Stutzer A (2002). What can economists learn from happiness research?. J Econ Lit.

[21] Ghebreyesus TA (2017). All roads lead to universal health coverage. Lancet Glob Health.

[22] Wherry LR, Kenney GM, Sommers BD (2016). The role of public health insurance in reducing child poverty. Acad Pediatr.

[23] Flavin P (2018). State Medicaid expansion and citizens’ quality of life. Soc Sci Quart.

[24] Tran NLT, Wassmer RW, Lascher E (2017). The health insurance and life satisfaction connection. J Happiness Stud.

[25] Finkelstein A, Taubman S, Wright B, Bernstein M, Gruber J, Newhouse J, Allen H, Baicker K (2012). The Oregon health insurance experiment: evidence from the first year. Q J Econ.

[26] Keng S-H, Wu S-Y (2013). Living happily ever after? The effect of Taiwan’s national health insurance on the happiness of the elderly. J Happiness Stud.

[27] Liao P-A, Chang H-H, Sun L-C (2012). National health insurance program and life satisfaction of the elderly. Aging Ment Health.

[28] Chiao C, Ksobiech K, Wei C-Y (2014). National health insurance and life satisfaction in late life: longitudinal findings from a natural experiment in Taiwan. J Public Health.

[29] Blanchflower D. Happiness and health care coverage. IZA Discussion Paper No. 4450, Institute of Labor Economics; 2009. https://www.iza.org/publications/dp/4450/happiness-and-health-care-coverage.

[30] Steptoe A (2006). Depression and physical Illness.

[31] Inglehart R, Haerpfer C, Moreno A, Welzel C, Kizilova K, Diez-Medrano JM, Lagos P, Norris E, Ponarin B, Puranen, et al. World values survey: all rounds–country-pooled datafile. Madrid & Vienna: JD Systems Institute & WVSA Secretariat; 2020. https://www.worldvaluessurvey.org/WVSDocumentationWV6.jsp.

[32] Schlenker BR, Chambers JR, Le BM (2012). Conservatives are happier than liberals, but why? Political ideology, personality, and life satisfaction. J Res Pers.

[33] Appleton S, Song L, Xia Q (2014). Understanding urban wage inequality in China 1988– 2008: evidence from quantile analysis. World Dev.

[34] Kollamparambil U (2020). Happiness, happiness inequality and income dynamics in South Africa. J of Happiness Stud.

[35] Neira I, Lacalle-Calderon M, Portela M, Perez-Trujillo M (2019). Social capital dimensions and subjective well-being: a quantile approach. J Happiness Stud.

[36] Fang Z (2017). Panel quantile regressions and the subjective well-being in urban China: evidence from RUMiC data. Soc Indic Res.

[37] Borah B, Basu A (2013). Highlighting differences between conditional and unconditional quantile regression approaches through an application to assess medication adherence. Health Econ.

[38] Niimi Y (2018). What affects happiness inequality? Evidence from Japan. J Happiness Stud.

[39] Becchetti L, Massariy R, Naticchioni P (2014). The drivers of happiness inequality: suggestions for promoting social cohesion. Oxf Econ Pap.

[40] Firpo S, Fortin N, Lemieux T (2018). Decomposing wage distributions using recentered influence function regressions. Econom.

[41] World Economic Situation Prospects and United Nations, Department of Economic and Social Affairs. World Economic Situation Prospects. 2014. https://www.un.org/en/development/desa/policy/wesp/wesp_current/2014wesp_country_classification.pdf. Accessed 20 Apr 2022.

[42] Welzel C (2013). Freedom rising human empowerment and the quest for emancipation.

[43] De Neve J, Krekel C. Cities and happiness: a global ranking and analysis. World Happiness Report; 2020. https://worldhappiness.report/ed/2020/cities-and-happiness-a-global-ranking-and-analysis/. Accessed 15 June 2022.

[44] Paprotny D (2020). Convergence between developed and developing countries: a centennial perspective. Soc Indic Res.

[45] King N, Harper S, Young M (2013). Who cares about health inequalities? Cross-country evidence from the world health survey. Health Policy Plan.

[46] Rothstein B, Uslaner E (2005). All for all: equality, corruption, and social trust. World Polit.

[47] Pickett K, Wilkinson R (2015). Income inequality and health: a causal review. Soc Sci Med.

[48] World Health Organization and International Bank for Reconstruction and Development/The World Bank. Tracking universal health coverage: 2017 global monitoring report. 2017. https://apps.who.int/iris/bitstream/handle/10665/259817/9789241513555-eng.pdf . Accessed 20 Apr 2022.

[49] Sarracino F, O’Connor K, Ono H. Making economic growth and well-being compatible: evidence from Japan. MPRA paper no. 93010. Munich Personal RePEc Archive; 2019. https://mpra.ub.uni-muenchen.de/93010/1/MPRA_paper_93010.pdf.

[50] Benning T, Alayli-Goebbels A, Aarts MJ, Stolk E, Ardine de Wit G, Prenger R, Braakman-Jansen L, Evers SMA (2015). Exploring outcomes to consider in economic evaluations of public promotion programs: what broader non-health outcomes matter most?. BMC Health Serv Res.

[51] Borghi J, Jan S (2008). Measuring the benefits of health promotion programmes: application of the contingent valuation method. Health Policy.

